# Fulminant Type 1 Diabetes Mellitus Caused by Drug Reaction With Eosinophilia and Systemic Symptoms (DRESS): A Case Report and Review of the Literature

**DOI:** 10.3389/fendo.2019.00474

**Published:** 2019-07-16

**Authors:** Bizhen Zhu, Jinzhun Wu, Guobing Chen, Yungang Yang, Cuili Yi

**Affiliations:** Department of Pediatrics, The First Affiliated Hospital of Xiamen University, Xiamen, China

**Keywords:** drug reaction with eosinophilia and systemic symptoms, drug-induced hypersensitivity syndrome, diabetes mellitus, autoimmune diseases, sequelae

## Abstract

Drug reaction with eosinophilia and systemic symptoms (DRESS), also known as drug-induced hypersensitivity syndrome (DIHS) is a rare, severe cutaneous adverse drug reaction characterized by fever, skin rashes, lymphadenopathy, leukocytosis with eosinophilia, and/or atypical lymphocytosis, and multiple visceral organ involvement. Moreover, patients with DRESS are at risk of developing autoimmune diseases including thyroiditis, diabetes mellitus (DM), and systemic lupus erythematosus (SLE), etc. several weeks or months after the initial resolution. We described a 9-month boy who was admitted to our hospital because of severe pneumonia and developed DRESS 3 weeks later. After the withdrawal of suspicious drug and administration of systemic corticosteroids, the patient's condition improved gradually. Nevertheless, hyperglycemia was detected 20 days after the initial onset of DRESS, and subsequent fulminant type 1 diabetes mellitus (F1DM) was diagnosed requiring continuous intravenous insulin infusion. After 13 months of follow-up, the blood glucose levels are now well-controlled. Literature research in PubMed for diabetes mellitus associated with DRESS showed 16 articles and 27 related case reports. Of 27 patients with DM related to DRESS, 11 were male, 16 were female. The mean age was 46 years. The duration from the onset of DRESS to the development of DM was 21 days on average. F1DM was diagnosed in 21 patients, T1DM was confirmed in 5 patients, and T2DM was only defined in 1 patient. Glutamic acid decarboxylase antibodies (GAD) were detected in 4 cases. Of 22 cases in which virus examination was carried out, evidence of virus reactivation was established in 16 cases (72.7%). Of patients with F1DM, 16 (88.9%) cases were evidenced by reactivation of herpes virus. A high frequency of HLA genotype and haplotype were found in 11 cases. DM was concomitant with acute pancreatitis in 3 patients and thyroiditis in 2 patients. No patients died from the disease. This work aims to raise awareness of long-term autoimmune sequelae in patients with DRESS.

## Introduction

The Drug Reaction with Eosinophilia and Systemic Symptoms (DRESS) is a rare but life-threatening adverse systemic reaction, which typically presents as extensive skin rashes, accompanied by fever, lymphadenopathy, hepatitis, hematologic abnormalities with eosinophilia and atypical lymphocytes, and various internal organ involvement. It was first described as a toxic reaction to phenytoin in 1938 (1). In the following several decades, it was named as Dilantin hypersensitivity, drug-induced lymphoma, and anticonvulsant hypersensitivity syndrome (2–4). The current term “drug rash with eosinophilia and systemic symptoms (DRESS)” was first proposed by Bocquet etal. in 1996 to distinguish it from other drug reactions that are not associated with eosinophilia (5). The “R” that initially represented rash in DRESS has been changed to reaction due to the variability of cutaneous manifestations. It is noteworthy that DRESS is also termed “Drug-induced hypersensitivity syndrome (DIHS)” by Shiohara etal., which emphasizes the association with human herpes virus 6 (HHV-6) reactivation (6). The clinical manifestation of DRESS ranges from mild skin rash with eosinophilia to fatal multi-organ dysfunction. The condition often has a relapsing-remitting course despite the withdrawal of drugs and is tightly associated with reactivation of various human herpes viruses, especially HHV-6. DRESS has a reported incidence of 1 in 10,000–100,000 new drug exposure (7). The characteristic features of this syndrome are the late onset, eosinophilia, and multi-systemic involvement. Another distinguishing feature is the possible persistence or worsening of symptoms, despite the discontinuation of the causative drugs. Limited studies showed that administration of corticosteroid might improve the outcome of patients with DRESS. Retrospective studies have described a 2–14% mortality rate from DRESS (8, 9). Although most patients will survive from the acute stage of DRESS, there is still a risk of developing autoimmune diseases several weeks or months after recovery from the syndrome, such as thyroiditis, diabetes mellitus (DM), and systemic lupus erythematosus (SLE), etc. Here, we present a case of fulminant type 1 diabetes mellitus (F1DM) in an infant after the resolution of DRESS.

## Report of a Case

A 9-month-old boy was admitted to the pediatric intensive care unit (PICU) due to tachypnea and cyanosis. Paroxysmal cough and wheezing were developed 6 days prior to admission. A few hours before hospitalization, he progressed to tachypnea and dyspnea. He was previously healthy except for an allergy to cefmenoxime. On admission, the body temperature was 36.9°C, pulse rate 172/min, respiratory rate 65/min, blood pressure 75/45 mmHg, and peripheral oxygen saturation 80%. Chest CT scan demonstrated disseminated infiltration and multiple consolidations in bilateral lung fields. Transthoracic echocardiography showed enlargement of the right ventricle and severe tricuspid regurgitation with an estimated pulmonary artery pressure of 85 mmHg. Severe pneumonia, acute respiratory failure, and pulmonary hypertension were diagnosed. Initial treatment encompassed empirical antibiotic therapy with meropenem (20 mg/kg Q8h) and azithromycin (10 mg/kg Qd), bosentan to lower pulmonary artery pressure, and tracheal intubation followed by mechanical ventilation.

After treatment, his condition improved and meropenem was degraded to piperacillin/tazobactam according to the laboratory findings on hospital day 6. Weaning from mechanical ventilation was successfully performed 2 weeks later. On day 19 of the hospitalization, he was transferred to the pediatric general ward.

**Figure 1:**
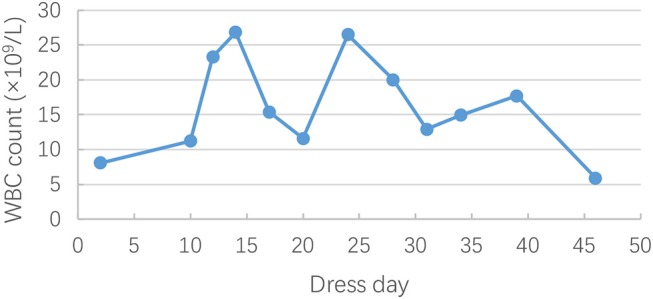
Changes in white blood cell (WBC) count during the clinical course of DRESS.

**Figure 2:**
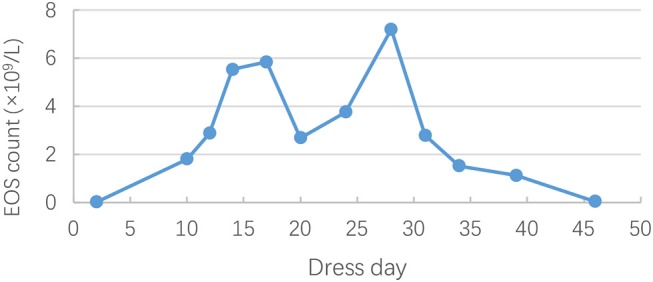
Changes in eosinophils (EOS) count during the clinical course of DRESS.

**Figure 3:**
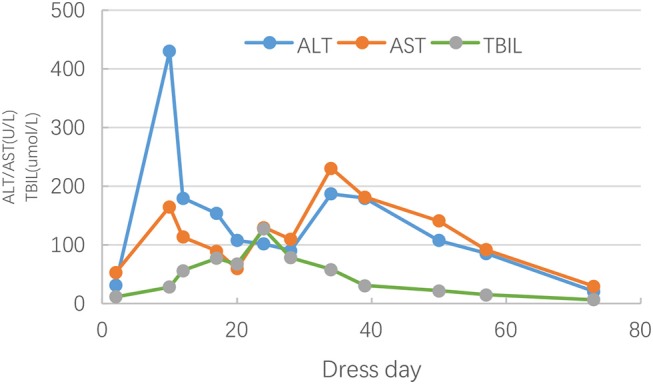
Changes in alanine aminotransferase(ALT), aspartate aminotransferase(AST), and total bilirubin(TBIL) during the clinical course of DRESS.

On hospital day 21 (general ward day 3), itchy maculopapular rash suddenly occurred in his face, then rapidly extended to trunk and extremities in association with facial swelling. There was no mucosal involvement. Cutaneous drug adverse reaction was suspected, and piperacillin/tazobactam was considered to be the culprit drug considering the previous allergy to cefmenoxime. Despite withdrawal of piperacillin/tazobactam, the rash worsened, and high-grade fever (39–40°C) turned up on hospital day 24. Antihistamine drugs and systemic steroids were administered. However, the rash progressed, and fevers persisted. Jaundice, hepatomegaly, bilateral cervical lymphadenopathy, and bilateral swelling of the parotid glands were observed on hospital day 30. Given the risk of progressing to a life-threatening condition, he was readmitted to PICU for further monitoring and management. Laboratory studies were remarkable for leukocytosis (26.82 × 10^9^/L [reference range, 4–10 × 10^9^/L]) (Figure 1) and eosinophilia (20.6% [reference range, 0–8%]) (Figure 2). The liver enzymes (Figure 3) were significantly elevated (aspartate transaminase 165 U/L [reference range, 9–50 U/L] and alanine aminotransferase 430 U/L [reference range, 15–40 U/L]). Total bilirubin surged to 127 umol/L [reference range, 5–25 umol/L] along with a level of direct bilirubin 100.8 umol/L [reference range, 0.2–7 umol/L]. Plasma albumin and fibrinogen decreased to 24.1 g/L (reference range, 40–55 g/L) and 0.85 g/L (reference range, 2–4 g/L), respectively. The plasma ammonia level elevated to 152 umol/L (reference range, 9–30 umol/L). Coagulopathy with a prolonged prothrombin time (24.9 s, [reference range, 10–16s]), a prolonged international normalized ratio [2.33, (reference range, 0.7–1.3)], and a prolonged activated partial thromboplastin time (70.6s, [reference range, 26–46s]) were also noted. The thrombocyte levels were normal. Blood culture and bacteriological tests were negative. DRESS was diagnosed based on RegiSCAR criteria. Piperacillin/tazobactam was supposed to be the most likely causative drug. Acute liver failure was also diagnosed according to the laboratory findings. Intravenous methylprednisolone (2 mg/kg^*^d) was continued, intravenous immunoglobulin (0.5 g/kg^*^4 day) was commenced, and plasma exchange was performed. His rash and liver damage gradually improved. Two weeks after onset, the initial skin rash began to vanish, and then he developed a severe exfoliative dermatitis followed by diffuse skin pigmentation.

**Figure 4:**
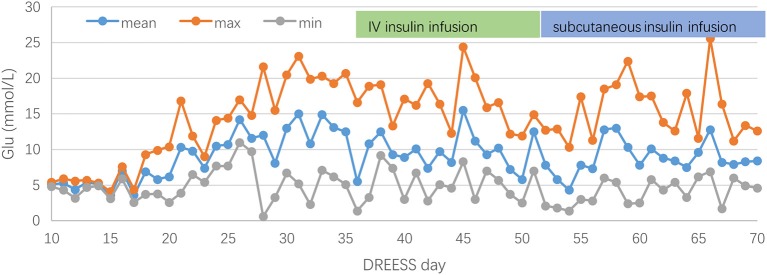
Fluctuations of blood glucose before and after insulin therapy during the clinical course of DRESS.

During his stay in PICU, blood glucose levels were monitored every 4 h and maintained within the normal range (4.3–6.9 mmol/L). On the 41st day of hospitalization, the boy was found hyperglycemic with a blood glucose level of 16.8 mmol/L. PH value, bicarbonate and base excess were normal. Stress hyperglycemia was suspected. Despite carbohydrates restriction in the diet and eliminating glucose from intravenous infusion, the blood glucose levels peaked up to 23.1 mmol/L necessitating continuous IV insulin infusion. Further laboratory studies showed a remarkable low fasting serum C-peptide (0.10 ng/ml, [reference range, 0.78–5.19 ng/ml]). Plasma amylase and lipase level were both normal. Thyroid function tests and antithyroglobulin antibody were normal, while antithyroid peroxidase antibody was positive (45.7 IU/mL, reference range, 0–30 IU/mL). Islet cell antibody (ICA), anti-insulin antibody (IAA), and anti-glutamic acid decarboxylase antibody (GAD) were negative. Abdominal ultrasonography showed no pancreatic abnormalities. Fulminant type 1 diabetes mellitus (F1DM) was confirmed. Blood glucose management was a huge challenge in the first few weeks, and the patient experienced several hypoglycemia events. Three weeks later, regular subcutaneous insulin injections of short-acting insulin analog were administered. Although the dose titration of insulin was rather tough, blood glucose levels gradually improved (Figure 4). Over the next month, the leukocyte, eosinophilia, hepatic enzyme levels, and coagulation factors were gradually normalized, and C-peptide finally became undetected (<0.01 ng/mL, [reference range, 0.78–5.19 ng/mL]). He experienced fluctuations of cutaneous symptoms during attempts at corticosteroid tapering, and eventually discontinued prednisone therapy 2 months later. After 13 months of follow-up, the patient still requires regular subcutaneous insulin injection, and the blood glucose levels are well-controlled. Besides, he had gained in weight by about 4 kg during 13-month follow-up.

## Review of the Literature

We gathered the clinical characteristics of diabetes mellitus (DM) associated with DRESS from literature published only in English language by accessing PubMed database from 1966 to 2018. The keywords used to search were DRESS, DIHS, and diabetes mellitus. A total of 16 articles and 27 related case reports were obtained between 2001 and 2018 (10–25). Their characteristics are summarized in Table 1. Of 27 patients with DM induced by DRESS, 11 were male, 16 were female. Onset age ranged from 5 years to 78 years. The mean age and median age was 46 years and 50 years, respectively. The interval between the onset of DRESS and the development of DM was 21 days on average. F1DM was diagnosed in 21 patients, T1DM was confirmed in 5 patients, and T2DM was only defined in 1 patient. The causative drugs were carbamazepine in 5 cases, mexiletine, minocycline, and allopurinol in 3 cases, diaminodiphenyl sulfone and dapsone in 2 cases, respectively. Islet-related autoantibodies testing was performed in 25 cases. Glutamic acid decarboxylase antibodies (GAD) were detected in 4 cases. Of 22 cases in which virus examination was carried out, evidence of virus reactivation was established in 16 cases (72.7%). Reactivation of HHV-6 was found in 13 cases (81.3%) among 16 cases performing HHV-6 tests. Coexistence of cytomegalovirus (CMV) and HHV-6 was observed in 3 cases. The concomitance of cytomegalovirus (CMV) and Coxsackie-B3 virus was found in 1 case. Unique coxsackie-B4 virus and CMV reactivation were seen in 1 case, respectively. Sixteen cases (88.9%) were evidenced reactivation of herpes virus in patients with F1DM. Of 16 cases in which HLA class I and II were analyzed, a high frequency of DRB1 was identified in 4 cases, a high frequency of B62 was found in 3 cases, DQA1 and DQB1 in 3 cases, and A24 in 1 case. DM was concomitant with acute pancreatitis in 3 patients and thyroiditis in 2 patients. No patients died from the disease.

**Table 1:** Reported cases of diabetes mellitus associated with DRESS.

**Source**	**Age, sex**	**Onset of DM after DRESS**	**Culprit drug**	**AA**	**Virus reactivation**	**HLA**	**Concomitant events**	**Underlying diseases**	**Type of DM outcome**
Sekine etal. (10)	77 year, F	14 d	Carbamazepine	ICA– GAD–	HHV−6+	DRB1 DQB1 DQA1	Cold agglutinin disease Pancreatitis	Postherpetic neuralgia	F1DM survival
Sommers etal. (11)	58 year, F	11 d	Allopurinol	/	/	/	Pancreatitis	CKD	F1DM survival
Seino etal. (12)	46 year, M	2 d	Mexiletine	GAD– ICA–	HHV−6+	DQA1 DQB1	Pancreatitis	T2DM	F1DM survival
Ozaki etal. (13)	50 year, M	120 d	Methimazole	GAD+	HHV−6+	/	–	Graves disease	T1DM survival
Chiou etal. (14)	21 year, M	60 d	Diclofenac Ibuprofen Penicillin,	GAD– ICA–	HHV−6+	/	–	Cough	F1DM survival
Zou etal. (15)	5 year, F	16 d	Phenobarbital	GAD– ICA–	EBV–	/	–	Epilepsy	T1DM survival
Brown etal. (16)	15 year, F	210 d	Minocycline	GAD+ IA2+	CMV– EBV– HSV1/2–	DRB1 DQA1 DQB1	Graves disease ANA/anti–Sm+ Anti–SSA+		T1DM survival
Chen etal. (17)	47 year, F	48 d	Dapsone	GAD–	/	/	–	Vasculitis Pulmonary TB	F1DM survival
Minegaki etal. (18)	71 year, F	0 d	Mexiletine	GAD– ICA–	HHV6+ CMV+	/	Hashimoto's thyroiditis ANA/anti-SSA+	Arrhythmia	F1DM survival
Dubois-Laforgue etal. (19)	68 year, F	120 d	Amoxicillin Clavulanic acid	GAD– IA2–	HHV6– CMV/EBV–	/		Bronchitis	F1DM survival
Erdem etal. (20)	14 year, F	7 d	Carbamazepine	GAD– ICA–	EBV– HPV1/2–	/	Lung involvement Microscopic hematuria	Epilepsy	T2DM survival
Lan etal. (21)	13 year, F	180 d	Minocycline	GAD+ ICA–	/	/	TPO+ Rapid-onset alopecia	Acne	T1DM survival
Marchese etal. (22)	43 year, M	0 d	Dapsone	GAD– ICA–	/	/	Acute interstitial Nephritis/ thyroiditis	AIDS	F1DM survival
Takeno etal. (23)	78 year, F	46 d	Carbamazepine	GAD– ICA–	Cox–B4+	HLA–A24	/	Blepharospasm	F1DM survival
Chiang etal. (24)	44 year, M	15 d	Unknown	GAD+	/	/	/	Hypertension, CHF/prediabetes	T1DM survival
Onuma etal. (25)	61 year, F	20 d	Mexiletine	GAD–	HHV6+	HLAB52/62 DR2/DR4	/	Rheumatic fever	F1DM survival
Onuma etal. (25)	63 year, M	14 d	Carbamazepine	GAD–	HHV6–	–	/	Alcoholism	F1DM survival
Onuma etal. (25)	19 year, F	35 d	Carbamazepine	GAD–	HHV6+	HLA–B62 DR9	/	Schizophrenia	F1DM survival
Onuma etal. (25)	31 year, F	21 d	Diaminodiphenyl sulfone	/	HHV6+	–	/	Livedo reticularis	F1DM survival
Onuma etal. (25)	60 year, F	24 d	Diaminodiphenyl sulfone	ICA–	CMV+ Cox–B3+	DR4 DRW12	/	Erythema nodosum	F1DM survival
Onuma etal. (25)	40 year, M	45 d	Allopurinol	ICA–	/	BW35 DR4/DRW8	/	Hyperuricemia	F1DM survival
Onuma etal. (25)	69 year, M	23 d	Allopurinol	GAD–	HHV6+ CMV+	–	/	Atrial fibrillation, hyperuricemia	F1DM survival
Onuma etal. (25)	56 year, M	199 d	Phenytoin	GAD–	HHV6– CMV+	–	/	Subarachnoid, hemorrhage	F1DM survival
Onuma etal. (25)	61 year, M	14 d	Zonisamide	GAD–	HHV6+	–	/	Cerebral, hemorrhage	F1DM survival
Onuma etal. (25)	19 year, M	89 d	Salazosulphapyridine	GAD–	HHV6+	DRB1	/	Drug addiction	F1DM survival
Onuma etal. (25)	57 year, F	13 d	Minocycline or Cefdynyle	GAD–	HHV6+	B44/B61 DRB1	/	Hepatitis C	F1DM survival
Onuma etal. (25)	72 year, F	20 d	Unknown	GAD–	HHV6+	B13/B62 DRB1	/	Atrial fibrillation	F1DM survival

*F, female; M, male; AA, autoantibody; HLA, human lymphocyte antigen; /, represents not available or unreported; –, represents negative; +, represents positive; GAD, glutamic acid decarboxylase antibodies; ICA, islet cell antibodies; IA2, protein tyrosine phosphatase antibody; HHV-6, human herpes virus 6; CMV, cytomegalovirus; EBV, Epstein-Barr virus; Cox, Coxsackie; HPV, human papilloma virus; HSV, herpes simplex virus; ANA, antinuclear antibodies; Anti-Sm, anti-Smith antibody; anti-SSA, anti-SSA antibodies; TPO, antithyroid peroxidase; TGA, antithyroglobulin antibodies; CKD, chronic kidney disease; Pulmonary TB, pulmonary tuberculosis; AIDS, acquired immune deficiency syndrome; CHF, congestive heart failure; F1DM, Fulminant Type 1 Diabetes; T1DM, type 1 diabetes mellitus T2DM, type 2 diabetes mellitus*.

## Discussion

DRESS is a rare, severe cutaneous adverse drug reaction characterized by fever, skin rashes, lymphadenopathy, leukocytosis with eosinophilia, and/or atypical lymphocytosis, multiple visceral organ involvement, and autoimmune sequelae. The pathogenesis of DRESS remains far from fully understood, but is hypothesized to be multifactorial, involving a combination of impaired drug detoxification pathways, abnormal immunological reactions, underlying viral reactivation, and genetic susceptibility (26). The mechanism of the development of autoimmune diseases after DRESS is also unclear. Some authors hypothesized that drug-induced inflammation in DRESS, especially the loss of Treg function occurring after the acute phase might contribute to the development of autoimmune sequelae (27). Viral reactivation has also been proposed as another important trigger of the subsequent autoimmune diseases.

Fulminant type 1 diabetes mellitus is reported to be a predominant autoimmune sequela secondary to DRESS, requiring early recognition and intervention. Kano etal. conducted a survey on sequelae of the underlying disease in patients with DRESS, and the prevalence of Fulminant type 1 diabetes (F1DM) was reported as 3.45% (5/145) (27). F1DM is a subtype of type 1 diabetes mellitus (T1DM) characterized by rapid onsetalong with absolute destruction of pancreatic β-cells and absence of islet-related autoantibodies (28). Several drugs have been associated with post-DRESS F1DM including carbamazepine, mexiletine, minocycline, allopurinol, diaminodiphenyl sulfone, and dapsone. In our case, piperacillin/tazobactam was suspected as the causative drug. The appearance of F1DM after initial resolution of DRESS suggests shared pathogenesis between these two conditions. In our literature review, of 18 patients with F1DM correlated to DRESS, 16 patients (88.9%) had evidence of reactivation of herpes virus. It appears that there is a strong association between viral reactivation and the development of F1DM. It is reasonable to hypothesize that viral infections might be involved in the pathogenesis of F1DM. The innate and adaptive immune responses to virus infection might lead to aggressive destruction of pancreatic β-cells (27). A high frequency of specific HLA genotype and haplotype were reported to be correlated to F1DM with DRESS, suggesting that the genetic factor might also be involved in this pathogenesis (25). Further investigation should be performed to clarify the role of genetic susceptibility in F1DM with DRESS. Interestingly, in our literature review, DM was also concomitant with autoimmune thyroid diseases including Graves's disease, Hashimoto's thyroiditis, and unclassified thyroiditis, which suggests the development of autoimmune diseases in patients with DRESS might share some pathogenesis with the autoimmune polyendocrine syndrome.

The average interval between the onset of DRESS and the emergence of F1DM was 21 days in our study. Nevertheless, the development of other autoimmune sequelae might even be postponed for several months or years. SLE and rheumatoid arthritis were, respectively, documented to develop in 3.5 years and 10 years after the onset of DRESS (29, 30). The physician might neglect the association between DRESS and autoimmune diseases due to the long interval. Thus, the occurrence of autoimmune diseases could be underestimated. It is of significant importance to perform a long-term follow-up for patients with DRESS. Early identification of the sequelae and corresponding management might be useful to improve the outcome. According to published literature, the longest interval between the onset of DRESS and appearance of DM was seven months. In the light of adverse consequences of diabetic ketoacidosis (DKA), it is reasonable to monitor blood glucose or HbA1c for at least 7 months after the initial resolution in patients with DRESS, especially those along with evidence of viral reactivation. Owing to the routine monitoring of blood glucose in PICU, we were able to recognize DM timely and avoid the occurrence of DKA. Even so, the management of DM was still a big challenge. It took us more than 1 month to maintain satisfying blood glucose levels.

The mainstay of the treatment is the withdrawal of culprit drugs and administration of corticosteroids (26). Timely withdrawal of culprit drug, and topical corticosteroids may be adequate for mild cases. However, Systemic corticosteroids have been routinely advocated in cases of moderate to severe disease, especially those with critical visceral involvement (9). Even though there seems to be a strong relevance between virus activation and F1DM after DRESS, it is still unknown whether antiviral treatment can improve the outcome.

## Conclusion

To our knowledge, this is the first case report of F1DM induced by DRESS in China. Moreover, we reviewed the related literature available in the PubMed database to make a better understanding of the characteristics of DM caused by DRESS. Further studies should be conducted to illustrate the exact mechanism and risk factors to achieve earlier recognition and intervention. Given the association between HHV-6 infection and F1DM, HHV-6 examination should be considered in patients with DRESS. Long-term follow-up of autoimmune sequelae is strongly recommended.

## Ethics Statement

This study was approved by the ethics committee of The First Affiliated Hospital of Xiamen University, and written informed consent was obtained from the parents of the participant for the publication of this case report and any potentially-identifying images/information.

## Author Contributions

BZ wrote the manuscript. JW and GC revised the manuscript. YY and CY contributed to the collection of the clinical data.

### Conflict of Interest Statement

The authors declare that the research was conducted in the absence of any commercial or financial relationships that could be construed as a potential conflict of interest.
